# Management of Acute Urinary Retention due to Bladder Outlet Obstruction in the Setting of Incarcerated Gravid Uterus

**DOI:** 10.1155/criu/1691627

**Published:** 2025-11-21

**Authors:** Carolyn M. Nguyen, Corrie DeGraffenreid, Gabriela E. Halder

**Affiliations:** ^1^Department of Obstetrics and Gynecology, University of Texas Medical Branch, Galveston, Texas, USA; ^2^Department of Obstetrics and Gynecology, University of Alabama, Birmingham, Alabama, USA

**Keywords:** acute urinary retention, bladder decompression, incarcerated gravid uterus

## Abstract

**Objective:**

An incarcerated gravid uterus (IGU) is a rare condition that occurs when a retroverted gravid uterus becomes trapped between the sacral promontory and pubic symphysis. If untreated, IGU could have devastating consequences including uremia, renal failure, sepsis, peritonitis, uterine wall necrosis/rupture, and ultimately maternal and/or fetal death. Our objective is to provide education on the presentation, diagnosis, and management of a rare diagnosis to assist clinicians in better recognizing and treating IGU.

**Methods:**

We present two cases of gravid females with acute urinary retention (AUR) in the setting of IGU. In this case series, we will discuss patient presentation, workup, and management of both IGU and AUR secondary to IGU.

**Results:**

The first patient represents delayed recognition of IGU and a protracted clinical course resulting in increased morbidity. The second case represents heightened recognition of IGU and appropriate, timely management.

**Conclusion:**

Prompt bladder decompression by urethral or suprapubic catheterization is the mainstay treatment for nearly all etiologies of AUR. In the case of AUR due to IGU, it is imperative to address bladder decompression prior to the reduction of IGU.

## 1. Introduction

IGU refers to the entrapment of the uterus in the pelvic cavity behind the sacral promontory. Potential causes are uterine anomalies, fibroids, pelvic adhesions, an anatomically retroverted uterus, or a deep sacral cavity with a prominent promontory. Although there are no direct causes of IGU, there is a strong correlation with retroverted uteruses, which may predispose women to the condition. Between the 12^th^ and 14^th^ weeks of pregnancy, the gravid uterus transforms from a pelvic to an abdominal organ, and a retroverted uterus will usually spontaneously correct to an axial position as the fundus rises out of the pelvis and falls forward [1]. If it does not correct itself, IGU may occur; however, it is a rare diagnosis occurring in only 1 in 3000–10,000 pregnancies [2].

As IGU further worsens with the progression of pregnancy, the cervix becomes more superiorly displaced, thereby compressing the urethra and lower bladder and causing bladder outlet syndrome and resultant AUR [2]. In a systematic review of IGU, the most common presentation was urinary complaints and lower abdominal pain [3]. We report two cases highlighting AUR in the setting of IGU, management of bladder outlet obstruction during pregnancy; review clinical presentations highly suspicious of IGU; and discuss the implications for urogynecologists.

## 2. Case 1

A 29-year-old G3P2 female at 13w6d gestation presented to the emergency department (ED) with a newfound complaint of AUR, incomplete voiding with urinary dribbling, and pelvic pain. Prior to presentation, she denied any history of urologic pathology, diabetes, neurological dysfunction, or IGU in prior pregnancies. Physical exam showed mild tenderness to palpation of the suprapubic region. CBC, CMP, and urinalysis were within normal range. Bladder scan showed greater than 200 mL of retained urine. The patient was straight catheterized with 800 mL urine output. After catheterization, the patient reported her pain resolved. The on-call OBGYN was consulted and recommended close follow-up due to the risk of IGU. She was discharged in stable condition with an indwelling urinary catheter free to drain. She was not placed on antibiotics at this time as she had close follow-up scheduled.

She presented to the obstetrics clinic two days later. An active voiding trial was performed by backfilling the bladder with 300 mL of saline. The catheter was removed, and the patient was able to empty her bladder. A referral for maternal–fetal medicine (MFM) ultrasound was placed to evaluate for IGU.

One day later, she returned to the ED with recurrent AUR. A Foley catheter was reinserted and drained 1 L of urine. On pelvic exam, the cervix was lower in the pelvis at −1 station without any tenderness. A transvaginal ultrasound (TVUS) was performed in the ED, showing an anteverted uterus and live intrauterine pregnancy. OBGYN was consulted, and the patient was admitted overnight for monitoring. There was suspected mild IGU, but manual reduction was not performed due to the patient's significant physical discomfort. A referral to urogynecology was placed due to recurrent AUR, and the patient was discharged with an indwelling Foley catheter. At 15w3d, the patient presented to the urogynecology clinic. A repeat pelvic exam revealed an anterior cervix without pain on bimanual exam and hypertonic contraction of pelvic floor muscles to palpation. An active voiding trial was repeated, but the patient was unable to void. The patient was taught how to intermittently self-catheterize and was given a referral for pelvic floor therapy.

**Figure 1:**
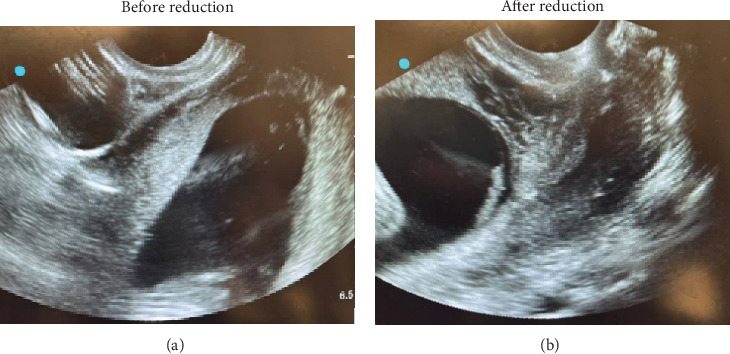
Bedside ultrasound imaging of the uterus. (a) Ultrasound reveals retroverted incarcerated gravid uterus. (b) After manual reduction resolving the incarceration (this study (Nguyen et al., 2025, “Management of Acute Urinary Retention due to Bladder Outlet Obstruction in the Setting of Incarcerated Gravid Uterus”. Case Reports in Urology)).

At 17w3d, the patient presented to MFM for TVUS. Imaging confirmed IGU (Figure 1). The patient was sent to labor and delivery (L&D) for manual reduction in the operating room under spinal anesthesia. Successful manual uterine reduction was performed, with postprocedure findings of the cervix in a neutral position (Figure 1). She was discharged on Hospital Day 1 without any further recurrence of urinary retention.

## 3. Case 2

A 39-year-old G2P1 female at 14 weeks gestation presented to the ED due to suprapubic and abdominal pain and AUR. Her past medical history included known 12 cm uterine fibroid. She had no history of urinary retention, diabetes, neurological dysfunction, or IGU in prior pregnancy. On exam, vitals were within normal range, and the abdomen was noted to be distended without tenderness. Labs obtained, including CBC, CMP, and urinalysis, were within normal range for pregnancy. TVUS was performed, with findings of live intrauterine pregnancy and large uterine fibroid measuring 8.2 × 6.0 × 10.9 cm. An indwelling Foley catheter was placed for bladder decompression, and the patient was discharged with a plan for follow-up with urogynecology in one day. She was not given prophylactic antibiotics at this time due to immediate follow-up. She presented to the urogynecology clinic the next day, and the Foley catheter was removed. An active voiding trial was performed with backfill of 300 cc of saline, and she was able to successfully void. On bimanual exam, the cervix was noted to be significantly anterior, and the sacral promontory was unable to be palpated posteriorly, with the suspected uterine body palpated over the normal position of the sacrum. Due to concern for uterine incarceration, the patient was referred to MFM. She was counseled on intermittent self-catheterization (ISC). She underwent TVUS with MFM the following day, with findings of IGU. She underwent successful manual reduction under spinal anesthesia at L&D.

## 4. Results

Both patients are without recurrence of IGU or AUR postoperatively after manual reduction. Both pregnancies resulted in successful spontaneous vaginal deliveries.

## 5. Discussion

There are currently no published guidelines for the management of IGU, with the primary literature consisting of literature reviews and case reports. It is imperative for physicians to be able to recognize, diagnose, and treat this rare condition at the risk of poor outcomes if not addressed adequately.

One of the most distinct symptoms is AUR in a gravid patient without any known inciting factors. We recommend IGU be at the forefront of the differential for AUR in a gravid patient due to the high morbidity if undiagnosed.

We propose bladder decompression with emergency catheterization as the initial treatment of AUR secondary to IGU. It is also reasonable to consider ISC or long-duration urinary catheterization with or without antibiotic prophylaxis for urinary tract infection [4]. This can be done in the interim until reduction maneuvers are performed. Passive reduction maneuvers and manual reduction are associated with better maternal–perinatal outcomes if diagnosed earlier than 20 weeks [5].

In our presented cases, the patients had an indwelling catheter placed in the ED at initial presentation of AUR, with subsequent removal and teaching of ISC in the urogynecology clinic. We find this to be appropriate management with emergent placement of an indwelling catheter in a setting that cannot educate or provide supplies for ISC. We recommend prompt follow-up in a clinical setting that can remove the indwelling catheter to prevent infection and properly educate on ISC while working up the primary cause of AUR.

Pelvic examination should be performed, followed by imaging with ultrasound or MRI. Obtaining the appropriate imaging when there is a clinical suspicion of IGU is essential to confirm the diagnosis. Ultrasound is the gold standard imaging modality throughout pregnancy. Ultrasound findings of most IGU cases show an anteriorly displaced cervix extending upward, superior to the bladder and pubic symphysis, with the fetus located deep in the posterior portion of the pelvis. As a result, the cervix may be difficult to locate and identify on sonographic evaluation [6]. Therefore, we believe it is important to have personnel trained in obstetric imaging perform the ultrasound in a patient with suspected IGU. MRI has also emerged as a useful tool due to the larger and more panoramic view of the pelvis enabling a comprehensive view of anatomic relationships, which is important for the diagnosis of IGU [7]. As in the first presented case, due to confounding findings on ultrasound, diagnosis and treatment were delayed. Therefore, we suggest that if the overall clinical picture still indicates IGU, imaging can be escalated to MRI.

After identifying IGU as the reason for AUR, and if spontaneous reduction does not occur, manual reduction is recommended. The current consensus is for the obstetrician to attempt manual reduction before 20 weeks [8]. It is also recommended to empty the bladder with a catheter immediately prior to attempting manual reduction with uterine repositioning. We suggest neuraxial anesthesia during the maneuver for pain control and ease of manipulation of the uterus. If the maneuver is successful, at-home consolidation treatments can be done, including knee–chest, Trendelenburg positions, or use of a pessary. If the manual reduction maneuver fails, vaginal balloon or vaginal gauze packing are also effective treatments reported in case studies from Zhuang et al. [9] Lastly, more invasive interventions may be performed, including colonoscopy-assisted repositioning, laparoscopy, or laparotomy to relieve an IGU [5].

## 6. Conclusion

The cases presented in this limited series emphasize the importance of early physician recognition of IGU when AUR is the primary presenting complaint. Our first case had a delay in diagnosis due to nonrecognition on imaging and discrepancies in pelvic exams. The second case presented demonstrates appropriate diagnosis and timely treatment, an improvement noted due to physician knowledge of the condition at the time of presentation. Our study is limited by the small number of cases presented, but this again emphasizes the rarity of this condition. The strengths of our report include both patients' management with the same obstetric and urogynecology care teams, allowing for improvement of care on the second case presentation.

## Data Availability

Data sharing is not applicable to this article as no new data were created or analyzed in this study.
